# Left bundle branch–optimized cardiac resynchronization therapy: Pursuing the optimal resynchronization in severe (distal) conduction system disease

**DOI:** 10.1016/j.hrcr.2023.02.015

**Published:** 2023-02-26

**Authors:** Jesse Rijks, Justin Luermans, Kevin Vernooy

**Affiliations:** ∗ Department of Cardiology, Cardiovascular Research Institute Maastricht (CARIM), Maastricht University Medical Centre (MUMC+), the Netherlands; † Department of Cardiology, Radboud University Medical Centre (RadboudUMC), Nijmegen, the Netherlands

**Keywords:** Cardiac resynchronization therapy, Conduction system pacing, Device therapy, Heart failure, Left bundle branch area pacing

**Key Teaching Points**

• Stimulus–time to peak R wave in lead V_6_ can be longer than expected in left bundle branch area pacing (LBBAP) with left bundle branch capture due to severe distal conduction system disease, which can result in insufficient correction of dyssynchrony.
• In patients with nonselective left bundle branch pacing and severe distal conduction system disease, left ventricular (LV) septal myocardium and subsequent LV activation might be faster than LV activation via the (diseased) conduction system, in essence resulting in LV septal pacing only despite capture of the left bundle branch.
• Addition of an LV lead via the coronary sinus, resulting in left bundle branch–optimized cardiac resynchronization therapy, can be a good strategy to correct dyssynchrony when LBBAP alone fails to correct dyssynchrony.

## Introduction

Left bundle branch area pacing (LBBAP) has been introduced as an alternative pacing strategy to conventional biventricular cardiac resynchronization therapy (BiV-CRT).^1^^,^^2^ In addition to LBBAP alone, left bundle branch–optimized cardiac resynchronization therapy (LOT-CRT) has been introduced to further improve electrical resynchronization in cases in which LBBAP or BiV-CRT fails to sufficiently correct electrical dyssynchrony.^3^^,^^4^ LOT-CRT consists of LBBAP combined with an additional left ventricular (LV)–coronary sinus lead. In this case report, we describe a patient with severe (distal) conduction system disease in whom LOT-CRT might be specifically beneficial in addition to LBBAP alone.

## Case report

**Figure 1:**
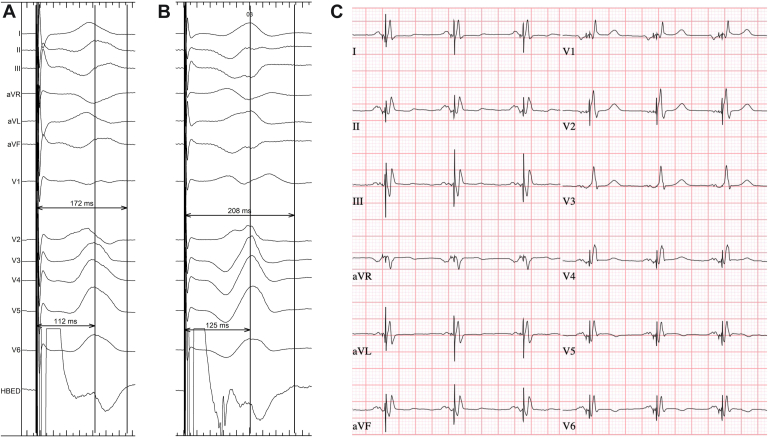
**A:** Nonselective left bundle branch pacing (LBBP) (5 V @ 0.4 ms) with stimulus–time to peak R wave in lead V_6_ (V6RWPT) 112 ms and paced QRS duration 172 ms. **B:** Selective LBBP (2 V @ 0.4 ms) with stimulus-V6RWPT 125 ms and paced QRS duration 208 ms. **C:** Final paced electrocardiogram. Left bundle branch–optimized cardiac resynchronization therapy with left bundle branch area pacing lead in the right ventricular (RV) port and left ventricle (LV)–coronary sinus lead in the LV port, programmed RV→LV + 40 ms, QRS duration 112 ms.

**Figure 2:**
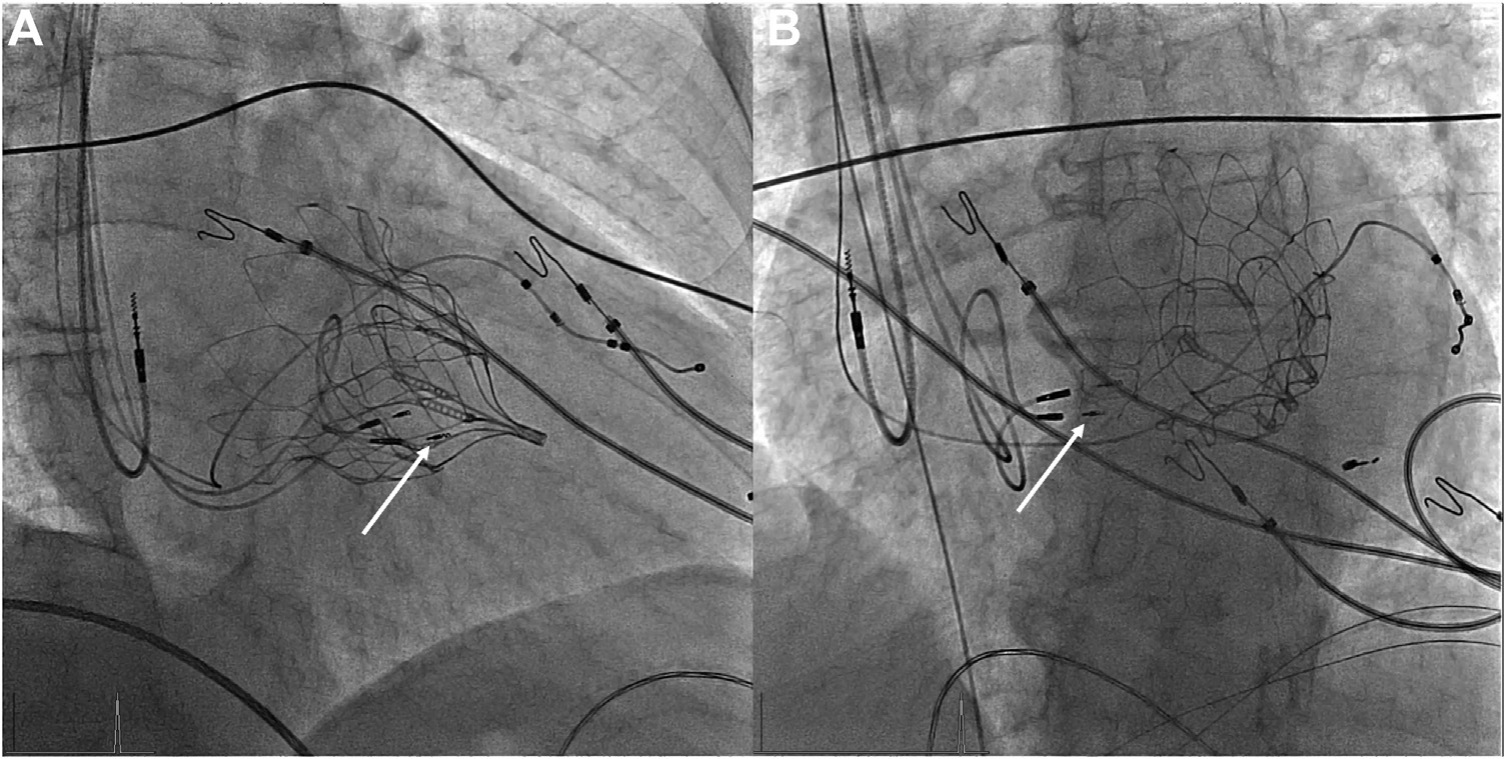
Final lead positions the right anterior oblique 20° **(A)** and left anterior oblique 30° **(B)** views. *White arrow* indicates the newly implanted left bundle branch area pacing lead.

A 72-year-old man with ischemic cardiomyopathy and a previous NeoChord (Neochord, Inc., Eden Prairie, MN) and Tendyne (Abbott Structural Heart, Santa Clara, CA) procedure for severe mitral regurgitation was referred to our device department. After implantation of a DDD pacemaker 2 years before referral with LBBAP because of second-degree AV block, his LV function had declined. Left ventricular ejection fraction (LVEF) decreased from 40% to 15%. The electrocardiogram recorded after initial implantation showed a paced qR morphology in lead V_1_ with a stimulus–time to peak R wave in lead V_6_ (V6RWPT) of 88 ms, without any criteria for left bundle branch capture. At follow-up, LBBAP seemed suboptimal, with a paced QRS duration of 185 ms with paced left bundle branch block (LBBB) morphology, possibly due to microdislodgment of the lead. We opted for implantation of a new LBBAP lead, with conventional BiV-CRT as bailout strategy. BiV-CRT was considered to be associated with increased risk because of a pseudoaneurysm compressing the coronary sinus as a complication of the previous NeoChord procedure. The new LBBAP lead was implanted according to the method previously described by Heckman et al.^5^ During implantation, a position at the left side of the interventricular septum was reached with a very long stimulus-V6RWPT of 112 ms, which usually is not indicative of left bundle branch capture (Figure 1A).^6^ Despite the long stimulus-V6RWPT, a morphology transition with high- and low-output pacing consistent with nonselective^6^ and selective^6^ left bundle branch pacing (LBBP) was found present. Stimulus-V6RWPT was longer with selective LBBP than with nonselective LBBP (125 and 112 ms, respectively) (Figures 1A and 1B). This long stimulus-V6RWPT might be due to severe distal conduction system disease. Moreover, in nonselective LBBP, stimulus-V6RWPT might be shorter because of faster activation of the LV septal myocardium and subsequent LV compared to activation via the (diseased) distal conduction system, in essence resulting in LV septal pacing only despite capture of the left bundle branch. Because of failure to correct the wide QRS with LV septal pacing alone, an additional LV–coronary sinus lead was implanted, opting for left bundle branch–optimized cardiac resynchronization therapy (LOT-CRT).^4^ Additional implantation of this LV–coronary sinus lead resulted in QRS duration of 112 ms (Figure 1C). A fluoroscopic image of the final lead positions is shown in Figure 2. The previously implanted LBBAP lead was abandoned and not extracted due to the risk of dislocating the newly implanted LBBAP and LV–coronary sinus leads, especially given that implantation of the LV–coronary sinus lead was difficult due to the pseudoaneurysm compressing the coronary sinus. At 6-month follow-up, LVEF improved to 40%, and New York Heart Association functional class improved from III to II.

## Discussion

With the availability of alternative pacing strategies such as LBBAP to conventional BiV-CRT in cardiac resynchronization therapy, patient selection is an important issue, even more so when both strategies can be combined with LOT-CRT.

BiV-CRT has proven to be an effective therapy in patients with heart failure and LBBB.^7^^,^^8^ LBBAP is considered an alternative to BiV-CRT because of its ability to correct LBBB, probably due to pacing beyond the site of conduction block.^9^ In patients with a typical LBBB pattern according to the criteria of Strauss et al,^10^ conduction block probably originates proximal in the left bundle branch and can be corrected by LBBAP alone. In these typical LBBB patients with a nonischemic cardiomyopathy, LBBAP has been shown to be associated with significant LVEF improvement, superior to that with BiV-CRT.^11^

LOT-CRT has been described as an alternative pacing strategy to classic BiV-CRT and LBBAP when these strategies alone result in suboptimal cardiac resynchronization therapy.^3^^,^^4^ This might be the case in patients in whom conduction block is not exclusively situated at the proximal part of the left bundle branch but severe (distal) conduction system disease is present. Here we present the case of a patient with persistent electrical dyssynchrony despite capture of the left bundle branch, indicating severe (distal) conduction system disease. LOT-CRT with addition of an LV–coronary sinus lead resulted in significant shortening of QRS duration and improvement in LVEF and symptoms.

## Conclusion

In this case report, we showed that stimulus-V6RWPT can be longer than expected in LBBAP with left bundle branch capture due to severe distal conduction system disease, resulting in LBBAP failing to correct dyssynchrony. In patients with nonselective LBBP and severe distal conduction system disease, LV septal myocardium and subsequent LV activation might be faster than LV activation via the (diseased) conduction system, in essence resulting in LV septal pacing only despite capture of the left bundle branch. LOT-CRT can be a good addition to LBBAP when the latter fails to correct electrical dyssynchrony.
